# Modern Myoma Treatment in the Last 20 Years: A Review of the Literature

**DOI:** 10.1155/2018/4593875

**Published:** 2018-01-24

**Authors:** Ahmed El-Balat, Rudy Leon DeWilde, Iryna Schmeil, Morva Tahmasbi-Rad, Sandra Bogdanyova, Ali Fathi, Sven Becker

**Affiliations:** ^1^Department of Gynecology and Obstetrics, Goethe-University, Frankfurt, Germany; ^2^Clinic of Gynecology, Obstetrics and Gynecological Oncology, Pius Hospital, University Hospital for Gynecology, Carl von Ossietzky University Medical School, Oldenburg, Germany

## Abstract

Myomas, also known as fibroids, are a specific characteristic of the human species. No other primates develop fibroids. At a cellular level, myomas are benign hyperplastic lesions of uterine smooth muscle cells. There are interesting theoretical concepts that link the development of myomas in humans with the highly specific process of childbirth from an upright position and the resulting need for greatly increased “expulsive” forces during labor. Myomas might be the price our species pays for our bipedal and highly intelligent existence. Myomas affect, with some variability, all ethnic groups and approximately 50% of all women during their lifetime. While some remain asymptomatic, myomas can cause significant and sometimes life-threatening uterine bleeding, pain, infertility, and, in extreme cases, ureteral obstruction and death. Traditionally, over 50% of all hysterectomies were performed for fibroids, leading to a significant healthcare burden. In this article, we review the developments of the past 20 years with regard to multiple new treatment strategies that have evolved during this time.

## 1. Introduction

Myomas or fibroids are the most common benign tumor of the female reproductive system, and while many remain asymptomatic, their impact on individual well-being can be significant [1, 2]. Traditionally, myomas have been the leading cause for hysterectomy, making this surgery the third most common surgical intervention worldwide [3, 4]. Removal of the uterus, while offering a definitive solution to the problem of fibroids, is inacceptable to women desirous of (further) childbearing or to some women simply because of psychological reasons. As a result, surgical myomectomy has been an alternative treatment option for over 100 years, originally by laparotomy and lately through minimal invasive techniques such as laparoscopy or hysteroscopy [5].

Any surgical intervention carries a small but real risk for complications: bleeding, possible need for transfusion, associated HIV and/or HCV-Infection, injury to bladder, bowel or ureters, subsequent adhesion-formation, complications of anesthesia and of hospitalization in general. Also, surgery requires a considerable infrastructure, including anesthesia, and remains cost-intensive.

Because of this, over the years conservative approaches that avoid surgery have been introduced, tested, reviewed, partially discarded, and partially accepted, leading to the currently available treatment options, as summarized in Table 1.

In this review we give updated information on the most recent literature to provide state of the art counseling to patients desirous for a thorough discussion of all available treatment options.

As increasing age during reproductive years, decreasing number of pregnancies, and increasing age of first pregnancy all lead to an absolute increase in myoma incidence, while increasing the number of women for whom hysterectomy is not an option; discussions about uterus-conserving interventions have been gaining momentum over the past 20 years [6].

This has subsequently lead to an increase in available uterine conserving treatment options.

## 2. Materials and Methods

A literature search was performed using Medline as the main resource. First, diagnosis-related keywords such as “myoma,” “fibroids,” “leiomyoma,” and “benign uterine tumors” were initially used, yielding between 5000 and 22000 hits (Tables 2 and 3). By comparison, “breast cancer” results in 337149 hits.

The first documented, and still available, article was published in 1887 by Dr. Thomas Keith in the British Medical Journal: “Results of Supravaginal Hysterectomy, with Remarks on the Old Way and the New of Treating Uterine Fibroids” [7]. It is a fascinating article and can only be recommended as a humbling experience with regard to how slow medical progress can truly be. Also, in the second sentence of the article, a mortality of 7.1% is cited without much comment. Therefore, on the other hand, there has been a lot of improvement.

Of particular interest is the second article on the subject, also from the British Medical Journal—German literature not having been scanned yet. It is from 1888 by Dr. W. J. Tivy about “Notes on Three Cases of Uterine Fibroids under Treatment by Apostoli's Electrical Method” [8]. Already the second available article in the English literature explores alternative treatment options.

The enthusiasm with which this novel—and now largely forgotten—technique is proposed puts the introduction of new treatment approaches into a historical perspective and underlines the need for some form or scientific evaluation. It is important to remember that the prospective randomized trial only became the standard of medical research after the Second World War.

In a second step, diagnosis and therapeutic keywords were combined: “myoma treatment,” “fibroid treatment.” These terms were further specified using terms such as “randomized trial,” “conservative,” “hormonal,” and “surgical.” A large part of available articles was not actually related to our subject matter or involved case reports. Our final selection included not only randomized trials, but also review articles, observational studies, and retrospective studies.

The available—and as always limited—literature that specifically offers prospective randomized data has been previously reviewed by the Cochrane Collaboration. It was our aim to present a balanced but clinically oriented review that focuses on real life data and relates to the everyday experience and the decision-making process surgical gynecologists face in their routine practice.

## 3. Results

### 3.1. Medical Treatment

While oral contraceptive pills have been used to treat myoma-related symptoms such as bleeding and dysmenorrhoea, their effect is usually based on their suppression/regulation of the menstrual cycle. The effect of ethinyl-estrogen/progesterone containing pills on myoma growth is less clear. Few authors mention an effect on myoma size. Increasingly, new insights into the molecular biological effects of hormones on leiomyoma cells are being investigated; however, so far no direct therapeutic consequences have emerged. [9, 10].

The same is true for the widely used levonorgestrel intrauterine devices, with the most commonly used being Mirena®. Again, mostly bleeding- and dysmenorrhoea-related symptoms are treated while the actual myoma size remains largely unchanged [11].

Thus, until recently conservative medical treatment focused on symptom control, which is appropriate for a disease that only rarely becomes life-threatening and tends to diminish after menopause. This approach of course does not address the problem of observing a potentially large fibroid uterus for another 40 years of life-expectancy after 50, when it increasingly becomes an undiagnosed complex solid pelvic tumor, which of course has implications in a 70-year-old women different from those in a 45-year-old women—particularly when a new doctor assumes the care and responsibility of watching a pathologic growth that has never been histologically evaluated.

Recently, selective progesteron-receptor modulators (SPRMs) such as asoprisnil, ulipristal, and telapristone have been evaluated as therapeutic agents for uterine myomas. [12]. The PEARL I and PEARL II trials have shown the ability of ulipristal acetate not only to control myoma-associated bleeding, but also to significantly decrease myoma size, though there is justified discussion as to how clinically significant this size reduction really is [13].

While ulipristal acetate is not yet available in the United States, it has been a considerable commercial success in Europe, where it is sold under the trade name of Esmya®. The success of this highly innovative medication is due not so much to its ability to decrease myoma size but to its ability to control bleeding symptoms without having many side effects. After the introduction of ulipristal acetate, the use of Gn-RH-analogs for the treatment of symptomatic fibroids, particularly the control of significant bleeding due to fibroids, has almost completely disappeared. Clearly, the known disadvantages of Gn-RH-analogs, that is, the severe postmenopausal-like side effects as well as the known negative effect on subsequent surgery, have led to a swift change in real life medical practice [14].

### 3.2. Surgical Treatment

Hysterectomy and myomectomy have been the treatment of choice for over 100 years; ever since surgery became safe and feasible. The historical articles mentioned in Material and Method underline this fact. Over the past 20 years, minimally invasive techniques have largely supplanted the open, laparotomic procedures. A large body of published literature has accompanied this technical process, providing scientific evidence of safety and superiority of the minimally invasive approach. Laparotomy is today practised on special clinical cases and in locations, where the necessary technology of laparoscopy is not readily available.

In this context, it is important to mention the sarcoma-morcellation discussion in the United States, which has the potential to roll back years of minimally invasive progress and lead to a recurrence of increased mortality and morbidity due to a return of laparotomy. While conflicting evidence is emerging, the fundamental question remains unanswered and controversial: Does mechanical morcellation affect the biological evolution of the underlying oncologic disease [15–17]? In the United States, for legal reasons, in-bag-morcellation techniques are introduced without a proper scientific evaluation of their complication rates and spilling. Overall, the entire morcellation discussion is clearly legally driven and has many similarities to the proposed association between silicone implants and autoimmune disease in the 1990s. For surgeons and patients a difficult situation has developed and the conclusion of the current discussion is not in sight. It is interesting to note that the possibility of occult sarcoma rarely comes up in association with the conservative treatment options, which, by definition, leave behind the uterine tumor without any diagnosis at all [18].

Key questions need to be answered during surgery for myoma and for hysterectomy. They are summarized in Table 4 and answered in the discussion section.

For the specific diagnosis of submucous, that is, intracavitary myomas, hysteroscopic myomectomy remains the only treatment option. Often, conservative treatment does not work in the long term, while the successful removal of an usually solitary submucous fibroid usually results in a complete resolution of all symptoms. While intramural and subserous myomas can be managed by “watchful waiting,” symptom-oriented treatment, or medical intervention (surgical or nonsurgical), the diagnosis of a submucous myoma as the cause of menorrhagia and dysmenorrhoea should lead to immediate scheduling of operative hysteroscopy.

### 3.3. Conservative, Nonmedical Treatment Options

Radiologically guided arterial myoma embolization was the first nonsurgical, nonmedical treatment approach to fibroid treatment. It was introduced in the late 90s, when no good treatment alternative existed and minimally invasive techniques had not yet become mainstream. At that time, removal of myomas usually meant open surgery, laparotomy, and the primary recommendation of most gynecologists for all women except those clearly desirous of future childbearing ability was hysterectomy.

Understandably, the disadvantages of arterial catheterisation in the groin when compared to laparotomy made this approach seem a viable alternative. [19]. The widespread introduction of minimally invasive surgical techniques leads to a reassessment of the clinical realities of myoma embolization: painful induced necrosis, often leading to unplanned hospitalizations, only very limited shrinkage of fibroids, unclear effect on childbearing, and subsequent need for additional surgical therapy (hysterectomy or myomectomy) [20]. Furthermore, radiation exposure has become an issue with many patients, which might explain why, after initial enthusiasm, this therapeutic approach has lost some of its appeal in recent years.

A novel technique that has found widespread acceptance only recently is the high frequency ultrasound treatment of fibroids, also known as HIFU. As a completely noninterventional treatment, it uses focused ultrasound waves to create thermic coagulation-zones within the myomas, again leading to subsequent necrosis and shrinkage. Two available technologies exist: the more widely used MRI-guided approach and the more advanced ultrasound-guided approach.

The question behind the scientific discussion about whether or not HIFU is an appropriate treatment for fibroids is more general: Does focused high-energy ultrasound have a true medical potential? Will it be the “knife” of the future surgeon? Already, publications of HIFU treatment of prostate cancer, breast cancer, and a variety of other benign or malignant tumors exist [21, 22].

There is little question that in selected patient populations, generally thought to be about 10% of all myoma-patients, HIFU can work. It will lead to necrosis and (partial) shrinkage of fibroids. Just like arterial myoma embolization, it is not an entirely benign procedure: the main complication is thermic injury to bowel, bladder, or, most commonly, the overlying skin. However, generally speaking the complication rate is very low. One disadvantage is the long treatment time, requiring patient to remain immobile in a specific position, sometimes for hours, with ultrasound-guided techniques requiring much less time [23].

## 4. Discussion

Modern myoma treatment has evolved over more than one hundred years. It involves traditional surgical methods that have been refined though new technological advances: minimally invasive, that is, laparoscopic myomectomy, novel medical treatments, that reflect our increasing understanding of the molecular biologic basics of fibroids as well as completely new approaches such as ultrasound treatment.

Important questions need to be discussed at a very individual level: symptoms, fertility, general attitude, expectations, and age, creating a multifactorial decision-matrix. The available evidence, as reviewed by this article, answers many scientific questions about effectiveness, side effects, long-term outcomes, and possible complications.

Few treatment options have been more thoroughly evaluated than myoma treatment, and, yet, no randomized prospective trial can answer the question: What is the best treatment? This question can only be answered as part of a shared patient-doctor decision-making process. One important aspect of this process is adequate counseling.  Table 5 summarizes the counseling-cascade that should be presented, discussed, and documented, to make sure that all options were truly presented to the patient.

The available literature clearly answers many questions. Are minimally invasive procedures safe? In the hands of a skilled surgeon, the answer is “yes.” Can nonsurgical interventions be recommended? Yes, they are safe and suitable for selected patients. Should hysterectomy be total or supracervical? Either approach can be chosen, it mostly depends on patient preference. No difference with regard to sexual function or pelvic floor support has been shown [24–27]. What is the upper size-limit for laparoscopic hysterectomy? It depends on the surgeon's willingness to torture him/herself and the OR team. Not everything that is laparoscopically possible makes laparoscopic sense. Should a salpingectomy always be performed during hysterectomy? Prospective data is lacking, but most gynecologic surgeons would counsel in favor of prophylactic salpingectomy [28]. Is surgery safer with or without in-bag morcellation? In the United States, in many hospital settings, unprotected morcellation is no longer allowed. Whether it is safer needs to be shown over the next years. Is there an upper limit for number of fibroids removed in laparoscopic myomectomy? Most surgeons would consider laparotomy when more than five fibroids are involved; however, the final decision depends on surgeon preference, site of the myomas, and patient desire to avoid laparotomy [29]. Which suture technique is superior: extracorporeal or intracorporeal. It depends on surgeon choice. Is intrauterine injection of vasoconstrictive drugs necessary? There is low quality evidence for its benefit [30] but most experienced surgeons will use it. Should the uterine arteries be routinely clipped in laparoscopic myomectomy? Clipping the uterine arteries requires fairly advanced technical skills. When serious bleeding is expected, it can make an extensive laparoscopic myomectomy laparoscopically feasible [31]. Should patient be pretreated with Gn-RH-analogs prior to hysteroscopic myomectomy? Ideally, visualization should be optimal during intracavitary procedures; that is, no endometrial lining should obstruct the surgeon's view. Gn-RH-analogs can help [32]. In summary, many ways lead to Rome as far as myoma treatment is concerned and surgery remains the most effective and the definitive highway.

## 5. Conclusion

Presently, the following options exist for effective myoma treatment, starting from the most conservative approach to the most invasive approach: symptomatic treatment with oral contraceptive pills or levonorgestrel-releasing IUDs, ulipristal acetate treatment, HIFU, myoma embolization, surgical myomectomy (hysteroscopic, laparoscopic, open), and hysterectomy. Different factors will affect the patient's choice: personal preference, age, desire for childbearing and future fertility, individual symptoms, and local medical availability of different treatment approaches. Because of the highly heterogeneous clinical situations, prospective randomized trials rarely reflect the individual patient-physician decision. At this point, no superior treatment can be defined. However, all treatment options included in this review have proven their safety and effectiveness and should be discussed with the patient, depending on their availability.

## Figures and Tables

**Table 1:** Treatment options for uterine myomas.

(1)	Oral contraceptive pills (symptomatic control of pain/bleeding)
(2)	Levonorgestrel-intrauterine device (IUD) (symptomatic control of pain/bleeding)
(3)	Ulipristal acetate treatment
(4)	Myoma embolisation by interventional radiology (induced ischemic myoma necrosis and shrinkage)
(5)	High frequency ultrasound treatment (induced thermic myoma necrosis and shrinkage)
(6)	Hysteroscopic myomectomy
(7)	Laparoscopic/open myomectomy and uterine reconstruction
(8)	Laparoscopic/open/vaginal hysterectomy

**Table 2:** Pubmed yield of different disease-specific keywords.

Fibroids	22332
Uterine fibroids	22052
Myoma	5408
Uterine myoma	22051
Leiomyoma	21001
Uterine leiomyoma	21001
Benign uterine tumors	5735

**Table 3:** Pubmed yield of different procedure specific keywords.

Myoma treatment	2611
Myoma treatment randomized trial	137
Conservative myoma treatment	121
Hormonal myoma treatment	126
Surgical myoma treatment	1599
Fibroid treatment	11555
Fibroid treatment randomized trial	487
Conservative fibroid treatment	333
Hormonal fibroid treatment	510
Surgical fibroid treatment	6724

**Table 4:** Important surgical questions.

(1)	Should hysterectomy be total or supracervical?
(2)	What is the upper size-limit for laparoscopic hysterectomy?
(3)	Should a salpingectomy always be performed during hysterectomy?
(4)	Is surgery safer with or without in-bag morcellation?
(5)	Is there an upper limit for number of fibroids in laparoscopy myomectomy?
(6)	Which suture technique is superior: extracorporeal or intracorporeal?
(7)	Is intrauterine injection of vasoconstrictive drugs necessary?
(8)	Should the uterine arteries be routinely clipped in laparoscopic myomectomy?
(9)	Should patient be pretreated with Gn-RH-analogs prior to hysteroscopic myomectomy?

**Table 5:** Treatment options, myoma counseling cascade.

(1)	Diagnostic evaluation: rule out submucous fibroid or nonmyoma diagnosis
(2)	Do nothing: watchful waiting
(3)	Only treat symptoms: bleeding, pain
(4)	Hormonal treatment: oral contraceptive pill, focus on bleeding
(5)	Hormonal treatment: ulipristal acetate
(6)	Fibroid embolization: radiology
(7)	HIFU treatment: radiology
(8)	Laparoscopic evaluation and surgical treatment
(9)	Laparoscopic evaluation and hysterectomy
